# *Blastocystis* spp. Positivity Is Associated with Altered Fecal Bacterial Communities and Reduced Short-Chain Fatty Acid Concentrations in Diarrheic Yaks

**DOI:** 10.3390/vetsci13090910

**Published:** 2026-09-04

**Authors:** Qiang Zhang, Ziye Zhu, Ziyi Xu, Muhammad Safdar, Wentong Liu, Kun Li

**Affiliations:** 1 Agriculture, Rural Affairs, Water Resources and Science & Technology Bureau of Lixian County, Aba Autonomous Prefecture 623100, China; 18090228046@163.com; 2 College of Veterinary Medicine, Nanjing Agricultural University, Nanjing 210095, China; 2024807227@stu.njau.edu.cn (Z.Z.); kyliam@stu.njau.edu.cn (Z.X.); 3 School of Basic Medicine, Hubei University of Arts and Science, Xiangyang 712000, China; 4 Department of Breeding & Genetics, Cholistan University of Veterinary and Animal Sciences, Bahawalpur 63100, Pakistan; msafdar@cuvas.edu.pk; 5 Institute of Molecular Biology and Biotechnology, Foundation for Research and Technology-Hellas, 70013 Heraklion, Greece

**Keywords:** 16S rRNA sequencing, *Bos grunniens*, intestinal dysbiosis, microbial fermentation, Qinghai–Tibetan Plateau

## Abstract

Yaks (*Bos grunniens*) are important livestock on the Qinghai–Tibetan Plateau, but the relationship between *Blastocystis* positivity, fecal bacterial communities, and microbial metabolites remains unclear. This cross-sectional study compared fecal bacterial profiles and short-chain fatty acid (SCFA) concentrations in six *Blastocystis*-positive and six *Blastocystis*-negative diarrheic yaks from Lhasa, Tibet Autonomous Region, China. Bacterial communities were characterized by 16S rRNA gene amplicon sequencing, and fecal SCFAs were quantified by gas chromatography–mass spectrometry. Compared with negative yaks, positive yaks had lower concentrations of acetic acid (*p* < 0.01), propionic acid (*p* < 0.01), butyric acid (*p* = 0.000381), valeric acid (*p* < 0.01), caproic acid (*p* = 0.00359), and isobutyric acid (*p* < 0.01), whereas isovaleric acid did not differ between groups (*p* = 0.310). A total of 2,751,028 raw sequences were generated, of which 2,620,949 passed quality filtering. ACE (*p* = 0.0068), Chao1 (*p* = 0.0071), Faith’s phylogenetic diversity (*p* = 0.0019), observed ASVs (*p* = 0.0064), Pielou’s evenness (*p* = 0.011), Shannon diversity (*p* = 0.008), and Simpson diversity (*p* = 0.037) were lower in positive yaks. The groups also differed in bacterial community composition, and several taxa previously associated with fiber degradation or SCFA formation had lower relative abundances in positive yaks. PICRUSt2 indicated differences in predicted microbial functional potential. These findings indicate that *Blastocystis* positivity was associated with altered fecal bacterial communities and reduced concentrations of several SCFAs in diarrheic yaks. Because of the small sample size, cross-sectional design, and potential confounding by diarrhea and other unmeasured factors, causal relationships cannot be established.

## Simple Summary

*Blastocystis* is a common intestinal microorganism detected in humans and animals, but its relationship with intestinal health in yaks remains unclear. This study compared fecal samples from six *Blastocystis*-positive and six *Blastocystis*-negative diarrheic yaks from Lhasa, China. Positive yaks had lower concentrations of several short-chain fatty acids and lower fecal bacterial diversity. Several bacterial groups previously associated with fiber degradation and short-chain fatty acid formation were also less abundant in the positive group. These findings indicate that Blastocystis positivity was associated with altered fecal bacterial communities and reduced fecal short-chain fatty acid concentrations. Because this was a small observational study involving only diarrheic animals, it cannot determine whether *Blastocystis* directly caused these differences. Larger controlled studies are required to determine the biological and veterinary significance of these associations.

## 1. Introduction

Yaks (*Bos grunniens*) are the principal livestock species of the Qinghai–Tibetan Plateau and are physiologically adapted to chronic hypobaric hypoxia, low temperatures, strong ultraviolet radiation, and seasonal forage scarcity. Approximately 17.5 million yaks are distributed worldwide, of which about 94.4% are raised in China [1]. Yaks provide meat, milk, fiber, hides, transportation, and dung fuel and are therefore essential to the livelihoods, food security, and cultural traditions of plateau pastoral communities [1]. Despite their adaptation to the high-altitude environment, nutritional stress and gastrointestinal diseases can compromise yak health and production. Recent microbiome investigations have shown that diarrhea is associated with disturbances in bacterial and fungal communities in yaks, highlighting the importance of maintaining intestinal microbial homeostasis in this species [2].

The intestinal microbiota comprises bacteria, archaea, fungi, viruses, and microbial eukaryotes that collectively contribute to nutrient metabolism, pathogen resistance, epithelial-barrier maintenance, immune regulation, and gut–brain communication [3,4]. These functions are particularly important in yaks because microbial fermentation supports the utilization of fibrous plateau forage. Short-chain fatty acids (SCFAs), principally acetate, propionate, and butyrate, are major products of microbial carbohydrate fermentation and contribute to epithelial energy metabolism, intestinal pH regulation, tight-junction integrity, and immune homeostasis [4]. Studies in yaks further indicate that changes in intestinal microbial communities are associated with antioxidant status, inflammatory mediators, and intestinal health [2,5]. However, the yak gut microbiota is also influenced by diarrhea, age, diet, grazing system, season, altitude, and management conditions; these factors must therefore be considered when interpreting associations between intestinal microorganisms and host health.

*Blastocystis* is a genetically diverse, anaerobic intestinal protist detected in humans and numerous domestic and wild animals [6,7]. Its cystic form can be transmitted through the fecal–oral route, including through contaminated water, feed, soil, and shared environments. Several subtypes have been detected in both humans and livestock, indicating potential—but not automatically confirmed—zoonotic transmission [6,7]. Molecular surveys have demonstrated substantial geographical variation in *Blastocystis* positivity among Chinese yaks. Reported prevalence values include 27.07% in Qinghai Province, 7.5% in free-ranging yaks sampled on the plateau, and 75.5% in Xinjiang [8,9,10]. A recent investigation in three counties of Lhasa reported prevalence values ranging from 80.0% to 90.7%, together with frequent detection of other enteric protozoa [11]. Differences among regions may reflect variation in climate, altitude, grazing practices, animal density, sanitation, sampling design, and molecular-detection methods. These findings demonstrate that *Blastocystis* is common in some yak populations and support further investigation of its relationship with intestinal health.

Nevertheless, the biological significance of *Blastocystis* colonization remains controversial and appears to vary according to host species, clinical condition, diet, microbial community, and subtype. In a large human cohort, *Blastocystis*-positive individuals showed greater bacterial alpha diversity and enrichment of taxa commonly associated with intestinal eubiosis [12]. Similarly, asymptomatic individuals carrying ST1 or ST2 exhibited greater microbial diversity and enrichment of health-associated bacteria [13]. In contrast, diarrheic patients carrying ST7 had lower bacterial diversity, altered community structure, increased Enterobacteriaceae and *Escherichia–Shigella*, and reduced *Bacteroides* and *Parabacteroides* compared with *Blastocystis*-negative diarrheic patients [14]. Subtype-dependent associations have also been observed in animals: ST10-positive forest musk deer showed greater bacterial richness and diversity, whereas ST5 positivity was not associated with a comparable diversity change [15]. Experimental evidence is similarly inconsistent; ST1-associated microbiota increased several SCFAs and promoted regulatory immune responses in a murine colitis model, whereas chronic ST4 colonization in rats was associated with altered microbiota and lower fecal SCFAs [16,17]. By comparison, most studies in cattle, sheep, goats, pigs, and poultry have focused on prevalence and subtype distribution rather than combined microbiota and SCFA profiling, leaving the metabolic consequences of *Blastocystis* positivity in domestic livestock insufficiently characterized [7]. These observations indicate that the effects of *Blastocystis* on intestinal microbial ecology and microbial metabolites cannot be predicted solely from parasite presence and may depend on the interaction between parasite characteristics, host physiology, intestinal disease status, and environmental conditions. In particular, whether the reduction or enrichment of SCFA-producing bacterial populations represents a consistent feature of *Blastocystis* colonization remains unresolved across different hosts. Therefore, comparative investigations integrating microbial community analysis and metabolite profiling are required to clarify the biological significance of *Blastocystis* in livestock species.

The conflicting findings among humans and other animal hosts suggest that the *Blastocystis*–microbiota relationship cannot be generalized across species or subtypes. Plateau yaks represent a distinctive host because chronic hypoxia, cold exposure, seasonal nutritional restriction, a high-fiber grazing diet, and specialized microbial fermentation may modify the interaction between intestinal protists, bacterial communities, and microbial metabolites. Unlike low-altitude domestic livestock and many human populations, plateau yaks have evolved under persistent environmental stressors that may influence intestinal microbial stability, fermentation capacity, and host–microbe interactions. The combination of high-altitude stress, specialized dietary adaptation, and susceptibility to gastrointestinal disorders provides a unique context in which protozoan colonization may have different consequences for microbial community structure and metabolic function. It was therefore hypothesized that the unique physiological and environmental characteristics of plateau yaks could be associated with a pattern of *Blastocystis*–microbiota interaction different from that reported in humans and low-altitude livestock. The central scientific question addressed in this study was whether *Blastocystis* positivity in diarrheic yaks was associated with altered fecal bacterial community structure, reduced microbial diversity, changes in predicted metabolic potential, and lower fecal SCFA concentrations. Accordingly, 16S rRNA gene amplicon sequencing and gas chromatography–mass spectrometry were used to compare bacterial profiles and SCFA concentrations between naturally *Blastocystis*-positive and *Blastocystis*-negative diarrheic yaks from Lhasa, Tibet Autonomous Region, China. However, despite the increasing recognition of *Blastocystis* occurrence in yak populations, the biological significance and potential mechanisms linking *Blastocystis* colonization with intestinal dysbiosis and metabolic alterations in plateau-endemic yaks remain poorly understood. Whether *Blastocystis* contributes to intestinal dysfunction through disruption of bacterial community structure and microbial metabolite production, particularly under high-altitude environmental conditions, remains an important unresolved question. Because the study was cross-sectional and involved naturally *Blastocystis*-positive animals, its purpose was to characterize associations rather than establish direct causation or a definitive pathogenic mechanism.

## 2. Materials and Methods

### 2.1. Animals and Sample Collection

This cross-sectional observational study was conducted using fecal samples collected from free-ranging yaks (*Bos grunniens*) maintained on a single farm in Lhasa, Tibet Autonomous Region, China, in 2025. Thirty fresh fecal samples were collected from diarrheic yaks directly from the rectum or immediately after defecation using sterile disposable materials. Samples were immediately placed on dry ice, transported to the laboratory, and stored at −80 °C until analysis. All 30 samples were screened for *Blastocystis* spp. Eleven samples were positive, and 19 were negative. Of the 11 positive samples, six yielded successful sequence confirmation and were therefore included as the *Blastocystis*-positive group for downstream bacterial-community and short-chain fatty acid (SCFA) analyses. Six *Blastocystis*-negative samples were selected from the 19 negative samples to provide an equal-sized comparison group. Thus, the final analyses included six *Blastocystis*-positive diarrheic yaks (bla group; bla1–bla6) and six Blastocystis-negative diarrheic yaks (cbl group; cbl1–cbl6). Both groups originated from the same farm and consisted of free-ranging diarrheic yaks; therefore, the cbl group represented a *Blastocystis*-negative diarrheic comparison group rather than a clinically healthy control group. Six negative samples were randomly selected. Detailed individual information on age, sex, body condition, dietary intake, vaccination history, recent antimicrobial or antiparasitic treatment, and diarrhea severity was not systematically available. Other enteric pathogens were not systematically investigated. These variables were therefore considered potential confounders when interpreting the results. Because only six animals were included in each group and no a priori power calculation was performed, the study was considered exploratory. All procedures were approved by the Ethics Committee of Nanjing Agricultural University (approval no. NJAU.No20240910164) and were performed in accordance with institutional animal-welfare guidelines.

### 2.2. Detection and Molecular Characterization of Blastocystis

Initial molecular screening identified 11 *Blastocystis*-positive samples among the 30 diarrheic yak fecal samples. Six of these 11 positive samples yielded successful sequence confirmation and were retained for downstream comparative microbiota and SCFA analyses.

Total genomic DNA was extracted from approximately 200 mg of each fecal sample using the Omega Fecal DNA Kit (Omega Bio-Tek, Norcross, GA, USA) according to the manufacturer’s instructions. A fragment of approximately 600 bp within the small-subunit ribosomal RNA gene of *Blastocystis* was amplified using primers Bs-RD5-F (5′-ATCTGGTTGATCCTGCCAGT-3′) and Bs-BhRDr-R (5′-GAGCTTTTTAACTGCAACAACG-3′), as previously described [11].

Each 25 µL PCR mixture contained 12.5 µL of 2× Taq PCR Master Mix (dNTPs 250 µM, MgCl_2_ 1.5 µM, Tris-HCl 10 mM, KCl 50 mM, Taq DNA polymerase 1.25 U/µL, Qiagen, Hilden, Germany), 1.0 µL of each primer at 10 µM, 1.0 µL of template DNA containing approximately 100 ng DNA (4 ng/µL), and 9.5 µL of nuclease-free water. Amplification consisted of initial denaturation at 95 °C for 5 min; 35 cycles of denaturation at 95 °C for 15 s, annealing at 59 °C for 15 s, and extension at 72 °C for 45 s; followed by a final extension at 72 °C for 10 min. The positive control produced an amplicon of the expected size, whereas no amplification was observed in the no-template control. PCR products were examined by electrophoresis on a 2% agarose gel. Amplicons of the expected size were purified using the QIAquick PCR Purification Kit (Qiagen, Hilden, Germany) and subjected to bidirectional Sanger sequencing by Eurofins Genomics (Ebersberg, Germany). The sequences were edited, assembled, and compared with reference sequences in the NCBI nucleotide database using BLAST (https://blast.ncbi.nlm.nih.gov/Blast.cgi?PROGRAM=blastn&PAGE_TYPE=BlastSearch&LINK_LOC=blasthome, accessed on 31 August 2026). The six sequence-confirmed samples showed 99.84% identity to GenBank accessions PP439446.1 and OR117711.1 and were deposited under accession numbers PX888047–PX888052. This high sequence identity supported identification of the recovered sequences as *Blastocystis* spp.; however, it was not considered sufficient to infer a specific subtype, pathogenic potential, or zoonotic transmission. Because definitive subtype assignment was unavailable, the sequences were reported as *Blastocystis* spp.

### 2.3. DNA Extraction, 16S rRNA Gene Amplification, and Sequencing

Microbial genomic DNA was extracted from approximately 200 mg of fecal material using the Omega Fecal DNA Kit according to the manufacturer’s instructions. DNA concentration and purity were assessed using a NanoDrop 2000 spectrophotometer (Thermo Fisher Scientific, Waltham, MA, USA), and DNA integrity was verified by electrophoresis on a 1.2% agarose gel.

The V3–V4 hypervariable region of the bacterial 16S rRNA gene was amplified using the universal primer pair 338F (5′-ACTCCTACGGGAGGCAGCAG-3′) and 806R (5′-GGACTACHVGGGTWTCTAAT-3′). Each 25-μL PCR reaction contained 5.0 μL of 5× FastPfu buffer, 2.5 μL of 2.5 mM dNTPs (0.1 mM), 1.0 μL of 5 μM of the forward and reverse primers (0.2 μM), 0.5 μL of FastPfu DNA polymerase (0.1 U/µL), approximately 10 ng of template DNA (0.4 ng/µL), and nuclease-free water to a final volume of 25 μL.

PCR amplification was performed under the following cycling conditions: an initial denaturation at 95 °C for 3 min; 30 cycles of denaturation at 95 °C for 30 s, annealing at 55 °C for 30 s, and extension at 72 °C for 45 s; followed by a final extension at 72 °C for 10 min. An extraction blank and a no-template PCR control were included throughout the workflow to monitor potential contamination.

PCR amplicons were verified by electrophoresis on a 2% agarose gel, purified using the AxyPrep DNA Gel Extraction Kit (Axygen Biosciences, Union City, CA, USA), and quantified using the QuantiFluor^®^-ST Fluorometer (Cat.# E6090, Promega, Madison, WI, USA). Purified amplicons were pooled in equimolar concentrations for library preparation using TruSeq Nano DNA LT Library Prep Kit (Illumina, San Diego, CA, USA). The amplicons were first fragmented to 300–500 bp, and then we proceeded with end repair, A-tailing, and adapter ligation. Later, the library was purified with BECKMANAM Pure XP beads (Beckman Coulter Life Sciences, Indy, IN, USA) and subjected to paired-end sequencing (2 × 300 bp) on an Illumina MiSeq platform (Illumina, San Diego, CA, USA) by Bioyi Biotechnology Co., Ltd. (Wuhan, China).

A total of 2,751,028 raw paired-end reads were generated, of which 2,620,949 high-quality reads were retained after sequence quality filtering and processing, corresponding to an overall read-retention rate of 95.27%. Sequencing quality was assessed using the standard Illumina MiSeq quality-control pipeline before downstream analysis. The average sequencing Q30 ratio of this research is 98.27%. No PCR amplicons were detected in the extraction blank or no-template control, confirming the absence of detectable contamination prior to sequencing.

### 2.4. Bioinformatic Analysis

Demultiplexed paired-end sequences were processed using QIIME 2 version 2023.9 [18]. Primer sequences and low-quality bases were removed, and sequence denoising, paired-end read merging, dereplication, error correction, and chimera removal were performed using the DADA2 plugin [19], which infers exact amplicon sequence variants (ASVs) rather than similarity-based operational taxonomic units (OTUs).

Representative ASV sequences were taxonomically classified using the q2-feature-classifier naïve Bayes classifier against the Genome Taxonomy Database (GTDB, release R214) reference database using the default confidence threshold (0.7), generating the GTDB-based taxonomic nomenclature (e.g., *Firmicutes_A*, *Firmicutes_D*, and *Arthrobacter_B*) reported throughout this study. Representative ASV sequences were aligned using MAFFT [20], and a phylogenetic tree was constructed for phylogenetic diversity analyses. Sequences assigned to chloroplasts, mitochondria, and other non-bacterial taxa were removed. Samples were rarefied to the minimum sequencing depth prior to downstream diversity analyses.

Alpha diversity was evaluated using the ACE, Chao1, Faith’s phylogenetic diversity, Observed Features, Pielou’s evenness, Shannon, Simpson, and Good’s coverage indices. Beta diversity was assessed using Bray–Curtis dissimilarity and visualized by principal component analysis (PCA), principal coordinate analysis (PCoA), non-metric multidimensional scaling (NMDS), and UPGMA hierarchical clustering. Differences in bacterial community composition between groups were evaluated using analysis of similarities (ANOSIM) with 999 permutations, and the R statistic and permutation-derived *p*-value were reported. Venn diagrams were generated to visualize shared and unique ASVs between experimental groups.

Heatmaps were generated from standardized taxon relative abundances. Relative abundances were transformed to Z-scores before visualization, and hierarchical clustering was performed using Euclidean distance and the complete-linkage algorithm.

Differentially abundant taxa were identified using Linear Discriminant Analysis Effect Size (LEfSe) [21]. Statistical significance was assessed using the Kruskal–Wallis test followed by pairwise Wilcoxon rank-sum tests, with *p* < 0.05 considered statistically significant. Taxa with a linear discriminant analysis (LDA) score ≥ 2.0 were considered differentially abundant. Additional comparisons of phylum- and genus-level relative abundances were performed using Student’s *t*-test or the Mann–Whitney *U* test, as appropriate according to data distribution.

Predicted microbial functional profiles were inferred using PICRUSt2 [22]. Predicted gene families were annotated against the MetaCyc and Kyoto Encyclopedia of Genes and Genomes (KEGG) databases. Functional predictions were interpreted as estimates of microbial functional potential derived from 16S rRNA gene profiles rather than direct measurements of microbial genes, gene expression, proteins, enzymes, or metabolic activity.

Raw sequencing data have been deposited in the NCBI Sequence Read Archive (SRA) under accession number PRJNA1414128.

### 2.5. Microbial Co-Occurrence Network Analysis

Genus-level co-occurrence patterns were examined using Spearman’s rank correlation. Genera detected in at least 50% of samples and having a mean relative abundance greater than 0.1% were retained. Correlations with an absolute Spearman coefficient of at least 0.70 and a Benjamini–Hochberg-adjusted *q*-value < 0.05 were included in the network.

Nodes represented bacterial genera, whereas edges represented statistically supported positive or negative associations. Node size was proportional to node degree, defined as the number of retained connections, and edge type indicated the direction of the correlation. Because of the small sample size and compositional nature of the sequencing data, network associations were regarded as exploratory and were not interpreted as evidence of direct biological interactions.

### 2.6. Fecal Short-Chain Fatty Acid Analysis

Fecal concentrations of acetic, propionic, butyric, isobutyric, valeric, isovaleric, and caproic acids were measured by gas chromatography–mass spectrometry using a method adapted from established fecal SCFA procedures [23,24].

Approximately 200 mg of thawed feces was homogenized in 1.8 mL of 0.5% (*v*/*v*) phosphoric acid (73 mM). Samples were vortexed thoroughly and centrifuged at 15,000× *g* for 10 min at 4 °C. A 400 µL aliquot of the supernatant was mixed with 400 µL of ethyl acetate, shaken for 30 min, and centrifuged again at 15,000× *g* for 10 min, and then filtered with via 0.22 μm PTFE syringe filter. An aliquot of the organic phase was supplemented with 4-methylvaleric acid at a final concentration of 1 mM as the internal standard and transferred to an autosampler vial. Calibration solutions containing all seven SCFAs and the internal standard concentration were prepared by stepwise serial diluting with 0.5% (*v*/*v*) phosphoric acid (73 mM) to generate seven point calibration working solutions.

Analyses were performed using an Agilent (Agilent Technologies, Santa Clara, CA, USA) 7890B gas chromatograph coupled to an Agilent 5977B mass-selective detector and equipped with an HP-FFAP capillary column (30 m × 0.25 mm internal diameter × 0.25 µm film thickness). A 1 µL aliquot was injected in splitless mode at an inlet temperature of 250 °C. Helium was used as the carrier gas at a constant flow rate of 1.0 mL/min.

The initial oven temperature was 50 °C and was maintained for 1 min, increased to 180 °C at 10 °C/min, and then increased to 240 °C at 20 °C/min and maintained for 5 min. Electron ionization was performed at 70 eV, and data were acquired in selected-ion-monitoring mode. SCFAs were identified by comparing retention times and characteristic ions with those of authentic standards. Calibration curves were generated from the analyte-to-internal-standard peak-area ratios, and a coefficient of determination of at least 0.99 was required. SCFA concentrations were normalized to wet fecal mass and expressed as µmol/g wet feces.

A pooled quality-control sample was analyzed after every ten study samples. Analytical performance was evaluated from calibration linearity, spike recovery, intra-day and inter-day precision, and limits of detection and quantification. The spike recoveries of all SCFAs in this study ranged from 96.71% to 102.37% with intra-day precision (RSD < 2.7%) and inter-day precision (RSD < 3.0%), and the limit of detection (LOD) and limit of quantification (LOQ) of all detected SCFAs in this research were 0.01–0.08 μg/mL and 0.05–0.26 μg/mL, respectively, which indicated satisfactory accuracy and negligible matrix interference of our results.

### 2.7. Statistical Analysis

Statistical analyses were performed using SPSS version 27.0 (IBM Corp., Armonk, NY, USA). Each individual yak was treated as an independent experimental unit. Where technical replicate measurements were available, replicate values were averaged before statistical testing so that each animal contributed a single value to the analysis.

Data distributions were evaluated using the Shapiro–Wilk test, and homogeneity of variance was assessed using Levene’s test. Variables meeting the assumptions of normality and equal variance were compared between the bla and cbl groups using an independent-samples Student’s *t*-test. Welch’s *t*-test was used for normally distributed variables with unequal variances, whereas non-normally distributed variables were compared using the Mann–Whitney *U* test. Normally distributed data are presented as the mean ± standard error of the mean, whereas non-normally distributed data are presented as the median and interquartile range.

All tests were two-sided, and *p* < 0.01 was considered statistically significant. For analyses involving multiple taxonomic and predicted functional comparisons, *p*-values were adjusted using the Benjamini–Hochberg false-discovery-rate procedure [25], with *q* < 0.01 considered statistically significant. Comparisons that did not remain significant after multiple-testing correction were regarded as exploratory.

## 3. Results

### 3.1. Molecular Detection of Blastocystis and Fecal SCFA Concentrations

Of the 30 diarrheic yak fecal samples screened, 11 were positive for *Blastocystis* by molecular screening. Six of these 11 positive samples yielded successful sequence confirmation and were included in the downstream comparative analyses. Their sequences showed 99.84% identity to GenBank reference sequences PP439446.1 and OR117711.1 and were deposited under accession numbers PX888047–PX888052. The sequence identity supported identification as *Blastocystis* spp.; however, definitive subtype assignment was not available.

**Figure 1:**
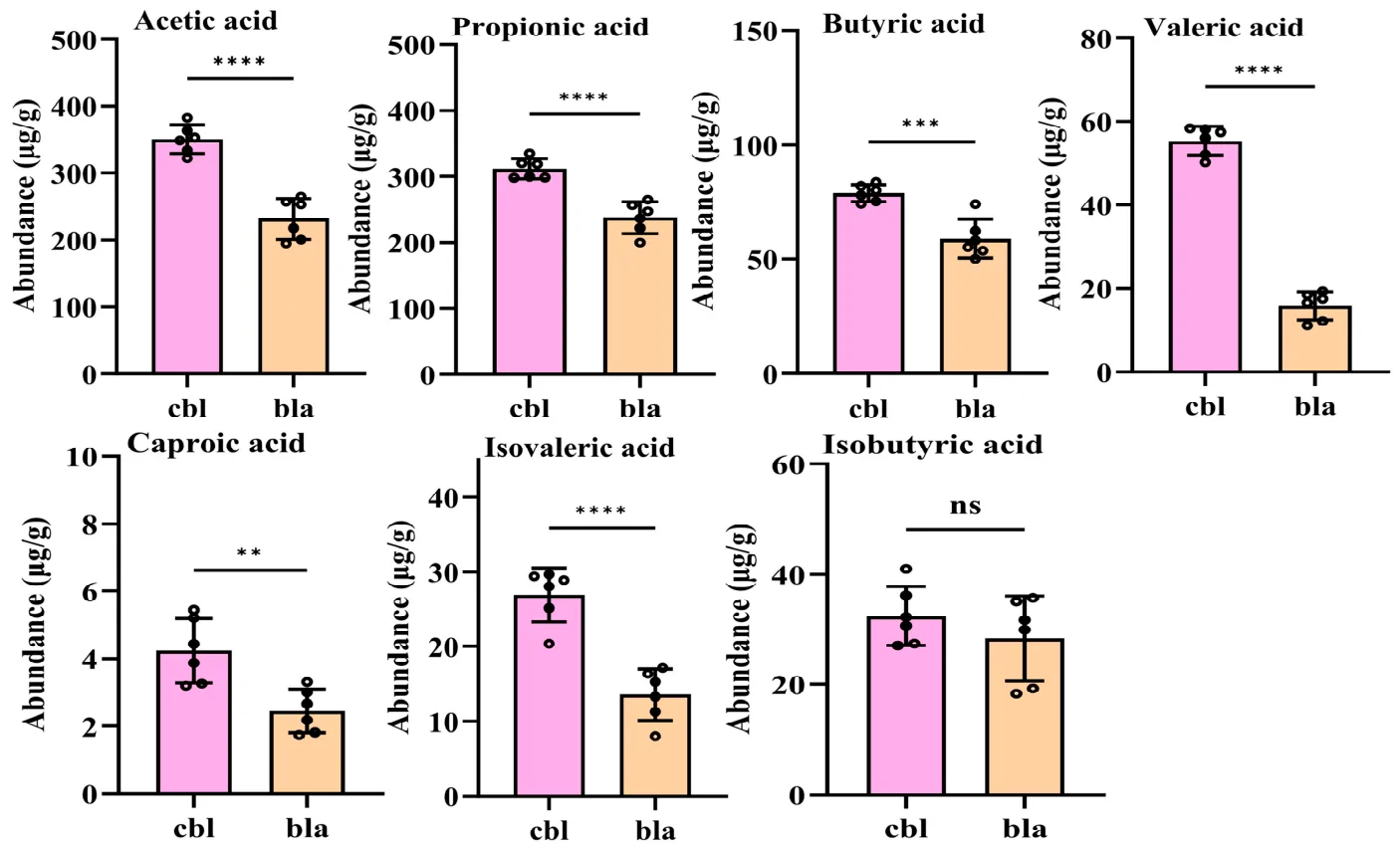
Fecal short-chain fatty acid concentrations in Blastocystis-positive and Blastocystis-negative diarrheic yaks. bla denotes Blastocystis-positive diarrheic yaks, and cbl denotes Blastocystis-negative diarrheic yaks (*n* = 6 per group). Bars represent the mean ± SEM, and points represent individual animals. Acetic, propionic, butyric, valeric, caproic, and isobutyric acid concentrations were lower in bla yaks, whereas isovaleric acid did not differ significantly. Between-group comparisons were performed using an unpaired Student’s *t*-test or Mann–Whitney *U* test, as appropriate. ** *p* < 0.01, *** *p* < 0.001, and **** *p* < 0.0001; ns, not significant.

Compared with the cbl group, the bla group had lower fecal concentrations of acetic acid by 34.0% (*p* < 0.01), propionic acid by 23.7% (*p* < 0.01), butyric acid by 25.2% (*p* = 0.000381), valeric acid by 71.4% (*p* < 0.01), caproic acid by 42.1% (*p* = 0.00359), and isobutyric acid by 49.7% (*p* < 0.01). Isovaleric acid did not differ significantly between the groups (*p* = 0.310; Figure 1). These findings indicate an association between *Blastocystis* positivity and lower concentrations of several fecal SCFAs in diarrheic yaks.

### 3.2. Sequencing Output, Alpha Diversity, and Bacterial Composition

**Figure 2:**
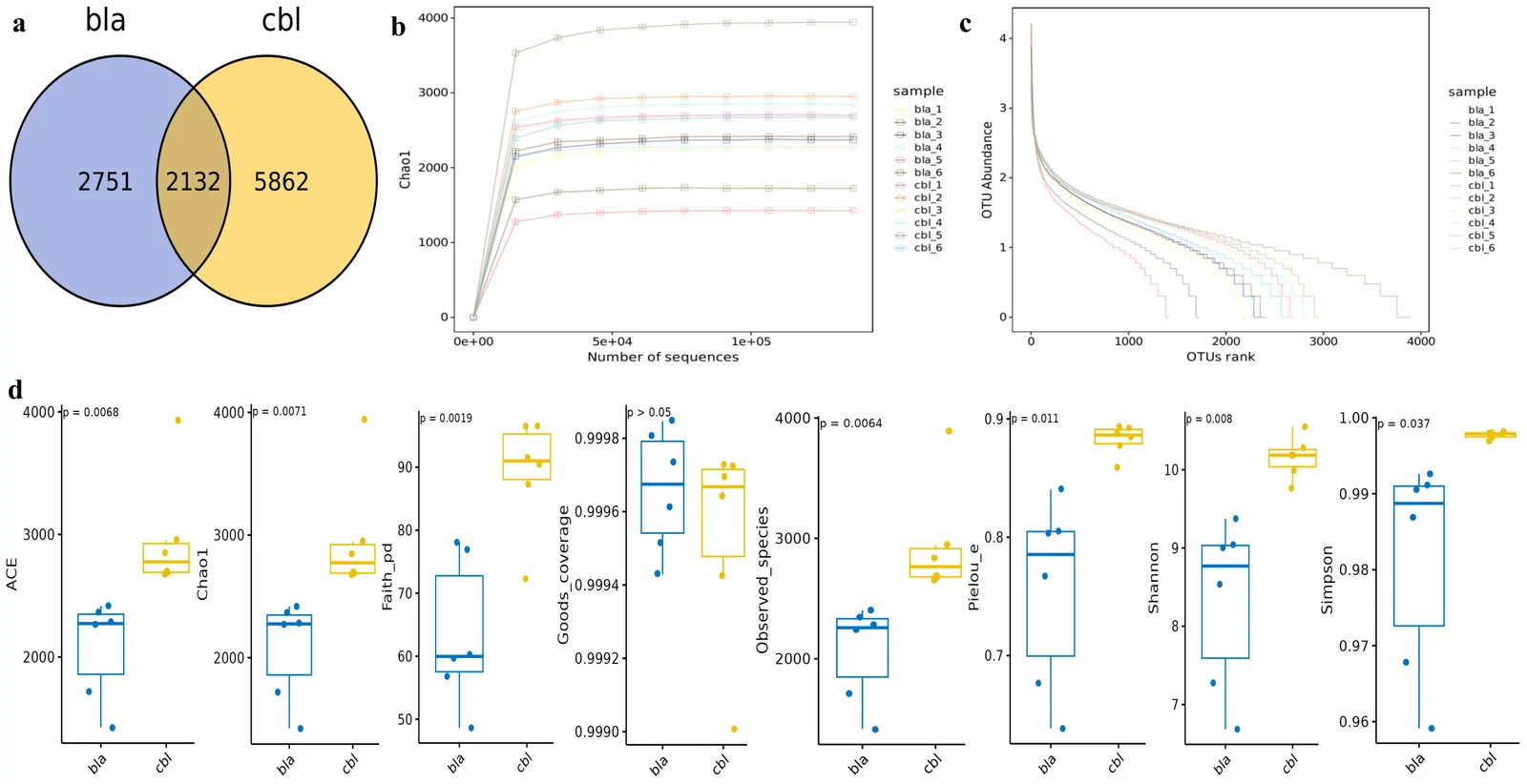
Amplicon sequence variant overlap, sequencing depth, and bacterial alpha diversity in *Blastocystis*-positive and *Blastocystis*-negative diarrheic yaks. Bla denotes *Blastocystis*-positive diarrheic yaks, and cbl denotes *Blastocystis*-negative diarrheic yaks (*n* = 6 per group). (**a**) Venn diagram showing ASVs unique to bla, unique to cbl, and shared between groups. (**b**) Rarefaction curves showing the number of observed ASVs as sequencing depth increased. (**c**) Rank-abundance curves showing bacterial richness and evenness. (**d**) ACE, Chao1, Faith’s phylogenetic diversity, Good’s coverage, observed ASVs, Pielou’s evenness, Shannon, and Simpson indices. Individual points represent animals. Boxes show the median and interquartile range, whiskers extend to 1.5 times the interquartile range, and points represent individual animals. *p* < 0.05 and *p* < 0.01.

A total of 2,751,028 raw sequences were generated from the 12 samples. After denoising, merging, and quality filtering, 2,620,949 sequences were retained, corresponding to an overall retention rate of 95.27% (Table 1). The sequences were resolved into 10,745 amplicon sequence variants (ASVs), including 2751 unique to the bla group, 5862 unique to the cbl group, and 2132 shared by both groups (Figure 2a). Rarefaction curves approached plateaus, indicating that sequencing depth was sufficient to characterize the bacterial communities (Figure 2b,c).

The bla group showed lower bacterial richness, phylogenetic diversity, and evenness than the cbl group. Blastocystis-positive yaks showed significantly reduced microbial richness and diversity compared with Blastocystis-negative yaks. Specifically, ACE, Chao1, Faith’s phylogenetic diversity, and observed amplicon sequence variants (ASVs) were significantly decreased (*p* < 0.01). Good’s coverage did not differ between the groups (*p* > 0.05; Figure 2d; Table 2).

**Figure 3:**
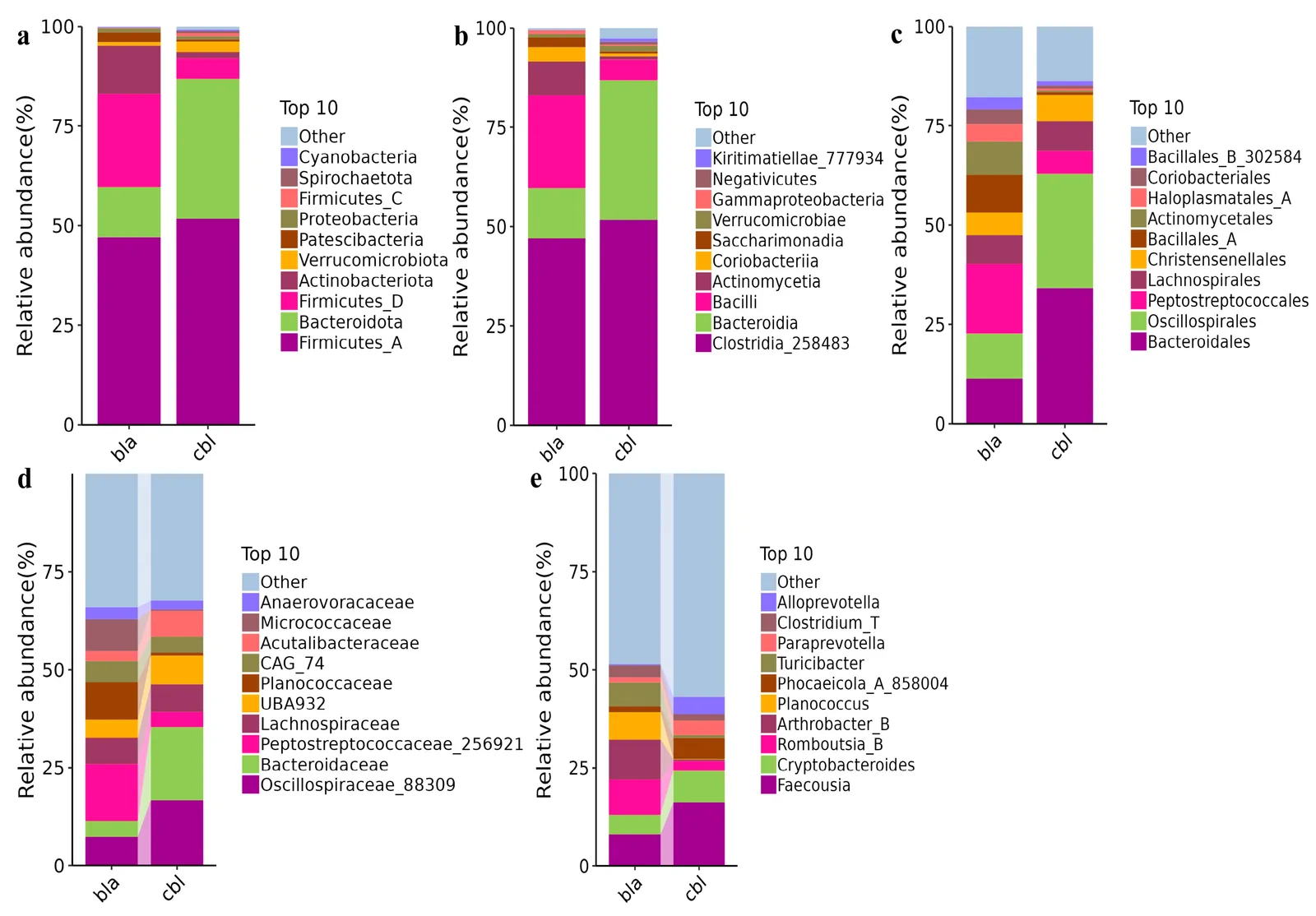
Fecal bacterial community composition in *Blastocystis*-positive and *Blastocystis*-negative diarrheic yaks. Bla denotes *Blastocystis*-positive diarrheic yaks, and cbl denotes *Blastocystis*-negative diarrheic yaks (*n* = 6 per group). Stacked bars show the mean relative abundances of the ten most abundant taxa at the (**a**) phylum, (**b**) class, (**c**) order, (**d**) family, and (**e**) genus levels. Taxa outside the ten most abundant categories are combined as “Other.” Values are expressed as percentages of total bacterial sequences.

At the phylum level, the principal bacterial groups were Firmicutes_A, Bacteroidota, and Firmicutes_D. Firmicutes_A accounted for 47.09% and 51.65% of sequences in the bla and cbl groups, respectively. Bacteroidota was less abundant in bla than cbl yaks (12.53% versus 35.19%), whereas Firmicutes_D was more abundant in bla than cbl yaks (23.36% versus 5.23%; Figure 3a).

**Figure 4:**
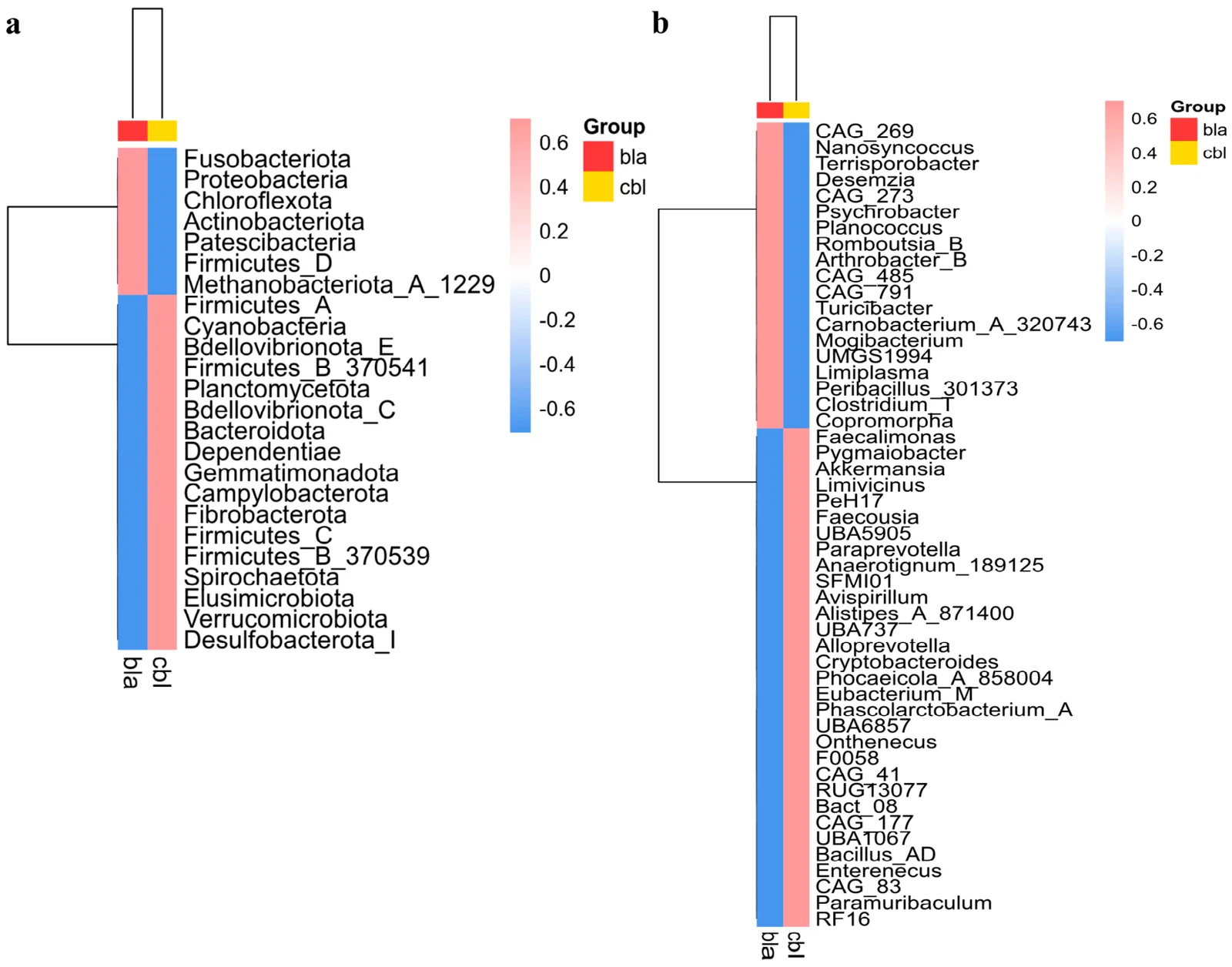
Heatmaps of fecal bacterial composition in *Blastocystis*-positive and *Blastocystis*-negative diarrheic yaks. Bla denotes *Blastocystis*-positive diarrheic yaks, and cbl denotes *Blastocystis*-negative diarrheic yaks (*n* = 6 per group). Heatmaps show standardized relative abundances at the (**a**) phylum and (**b**) genus levels. Red indicates comparatively higher standardized abundance and blue indicates lower standardized abundance. Dendrograms show hierarchical clustering of taxa and groups.

At the genus level, the most abundant taxa in the bla group were *Arthrobacter_B* (10.20%), *Romboutsia_B* (8.94%), and *Faecousia* (8.04%). In the cbl group, the predominant genera were *Faecousia* (16.17%), *Cryptobacteroides* (8.11%), and *Paraprevotella* (3.58%; Figure 3e). Heatmap analysis further showed higher relative abundances of Firmicutes_D, Actinobacteriota, and Patescibacteria in bla yaks, whereas Bacteroidota, Verrucomicrobiota, Firmicutes_C, Spirochaetota, Firmicutes_B_370539, and Desulfobacterota_I were more abundant in cbl yaks (Figure 4).

### 3.3. Beta Diversity and Differentially Abundant Taxa

**Figure 5:**
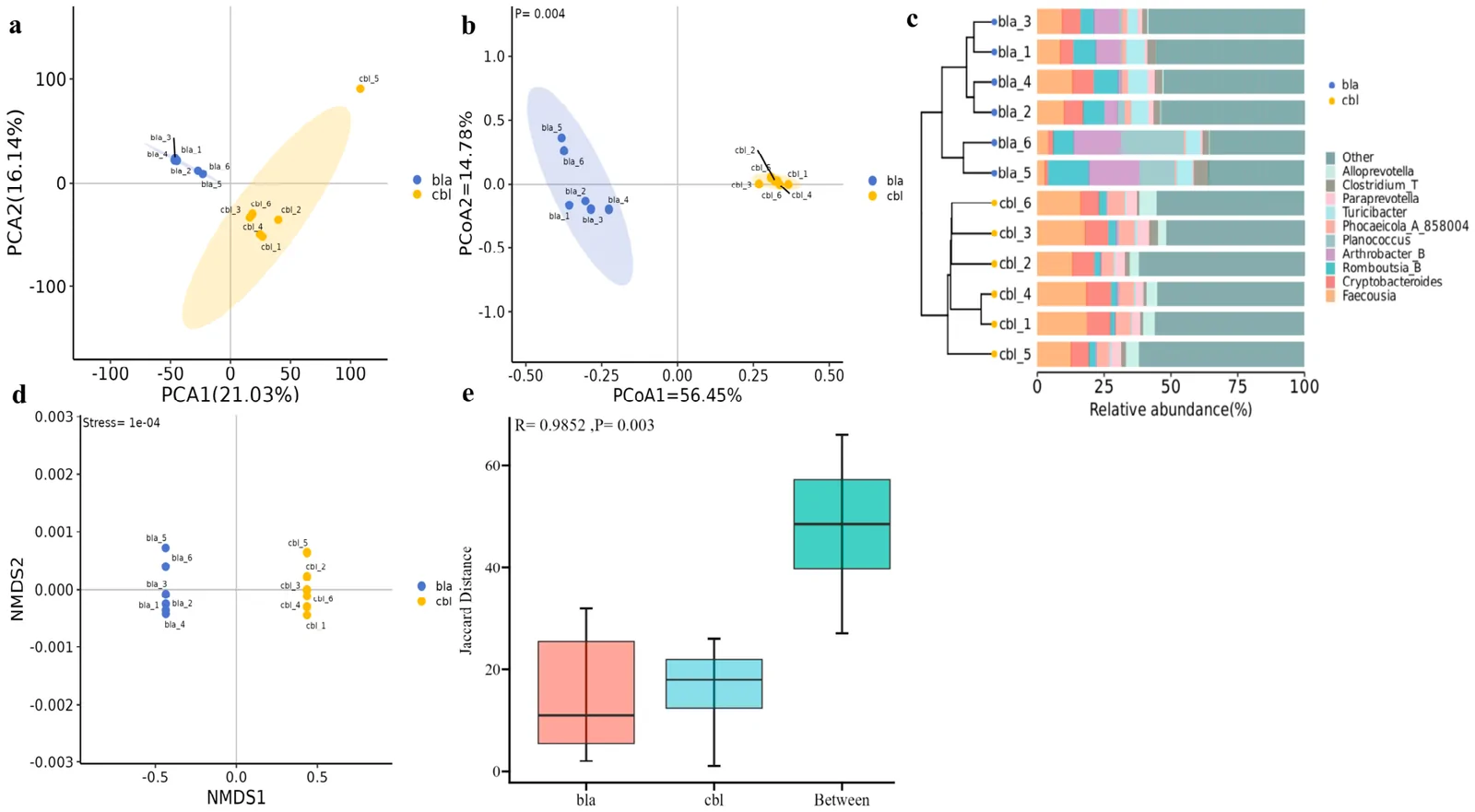
Beta-diversity analysis of fecal bacterial communities in *Blastocystis*-positive and *Blastocystis*-negative diarrheic yaks. Bla denotes *Blastocystis*-positive diarrheic yaks, and cbl denotes *Blastocystis*-negative diarrheic yaks (*n* = 6 per group). (**a**) PCA was performed based on bacterial community abundance profiles. (**b**) Principal coordinate analysis based on Bray–Curtis dissimilarity. Each point represents one animal, and shaded ellipses represent group dispersion. (**c**) UPGMA hierarchical clustering with genus-level bacterial composition. (**d**) Non-metric multidimensional scaling; the stress value indicates goodness of fit. (**e**) ANOSIM comparison of within-group and between-group distances; R = 0.9852 and *p* = 0.003.

PCA, PCoA, NMDS, and UPGMA analyses showed separation of the bacterial communities of bla and cbl yaks (Figure 5). The first two principal coordinates explained 56.45% and 4.78% of the total variation, respectively. NMDS yielded a stress value of 1 × 10^−4^, indicating an excellent representation of the Bray–Curtis distance matrix. ANOSIM demonstrated that between-group dissimilarity was significantly greater than within-group dissimilarity (R = 0.9852, *p* = 0.003).

**Figure 6:**
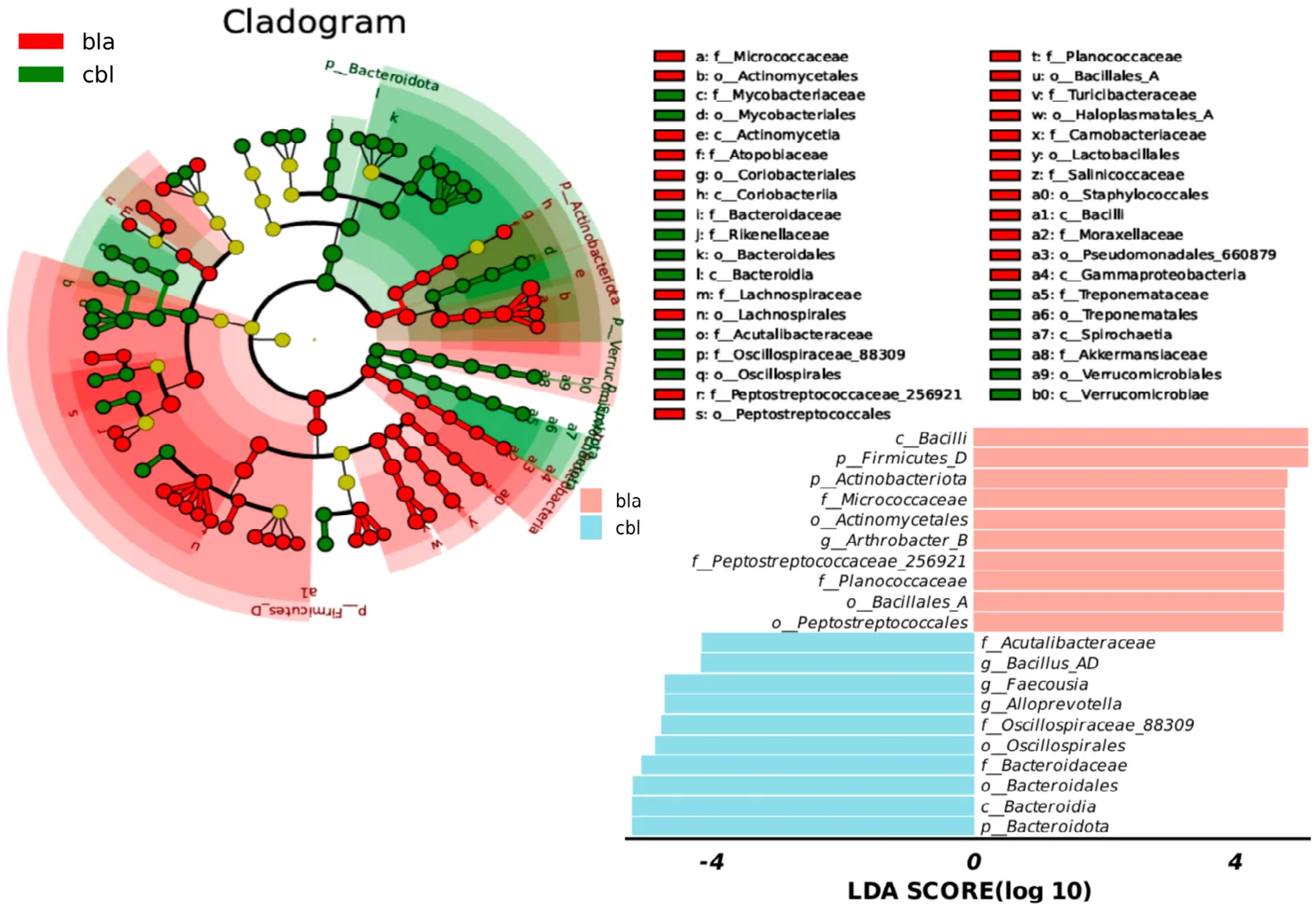
Differentially abundant fecal bacterial taxa identified by linear discriminant analysis effect size in *Blastocystis*-positive and *Blastocystis*-negative diarrheic yaks. Bla denotes *Blastocystis*-positive diarrheic yaks, and cbl denotes *Blastocystis*-negative diarrheic yaks (*n* = 6 per group). The cladogram displays differentially represented taxa from the phylum to genus levels. Node color indicates the group in which each taxon was more abundant, whereas yellow nodes indicate taxa not differing between groups. The bar plot shows taxa with a linear discriminant analysis score greater than 2.0. LEfSe significance thresholds were *p* < 0.05 for the Kruskal–Wallis and pairwise Wilcoxon tests.

LEfSe analysis identified Firmicutes_D, Actinobacteriota, Bacilli, Micrococcaceae, Planococcaceae, Bacillales_A, Peptostreptococcales, and *Arthrobacter_B* as the principal taxa associated with the bla group. Bacteroidota, Bacteroidia, Bacteroidales, Oscillospiraceae_88309, Spirochaetota, Verrucomicrobiota, *Faecousia*, and *Alloprevotella* were associated with the cbl group (Figure 6).

**Figure 7:**
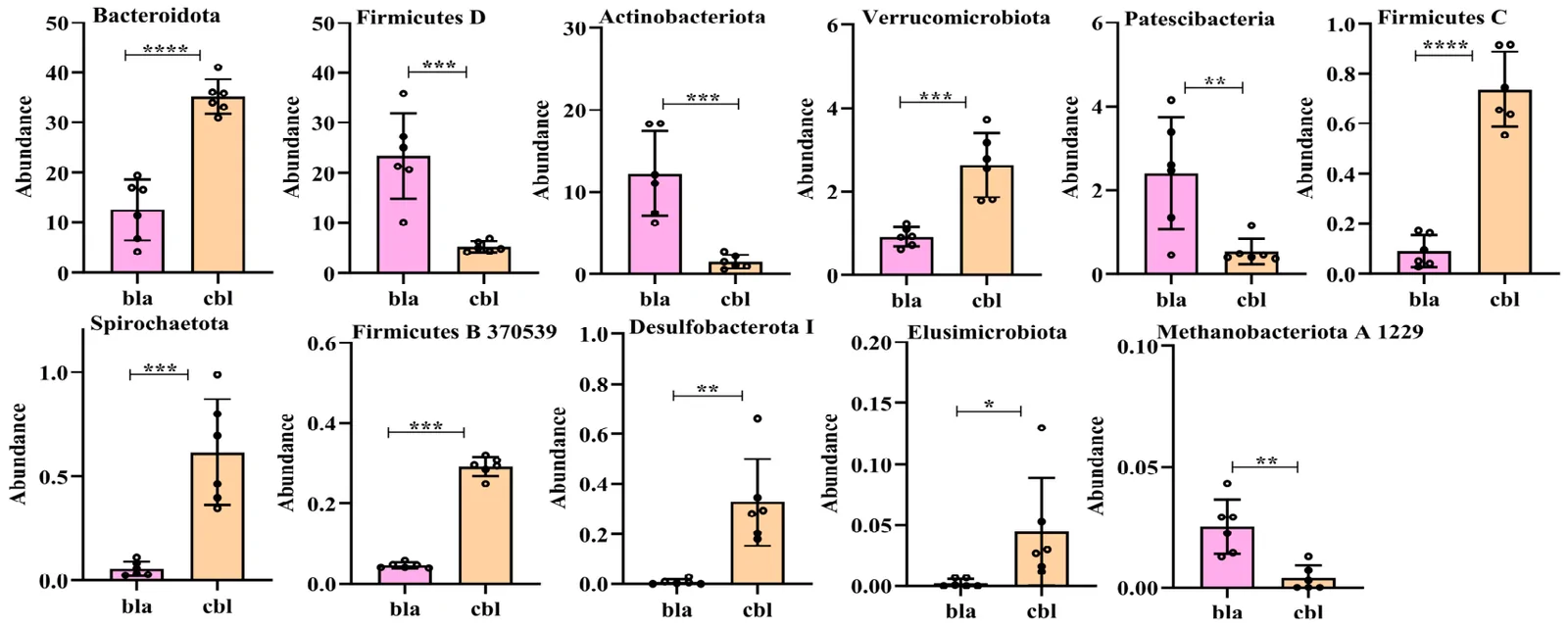
Relative abundances of bacterial phyla differing between *Blastocystis*-positive and *Blastocystis*-negative diarrheic yaks. Bla denotes *Blastocystis*-positive diarrheic yaks, and cbl denotes *Blastocystis*-negative diarrheic yaks (*n* = 6 per group). Bars represent the mean ± SEM, and points represent individual animals. The *y*-axis shows relative abundance as a percentage of total bacterial sequences. Between-group differences were evaluated using an unpaired Student’s *t*-test or Mann–Whitney *U* test, as appropriate. * *p* < 0.05, ** *p* < 0.01, *** *p* < 0.001, and **** *p* < 0.0001.

Exploratory phylum-level comparisons showed higher relative abundances of Firmicutes_D (*p* < 0.001), Actinobacteriota (*p* < 0.001), Patescibacteria (*p* < 0.01), and Methanobacteriota_A_1229 (*p* < 0.01) in bla yaks. In contrast, Bacteroidota (*p* < 0.0001), Verrucomicrobiota (*p* < 0.001), Firmicutes_C (*p* < 0.0001), Spirochaetota (*p* < 0.001), Firmicutes_B_370539 (*p* < 0.001), Desulfobacterota_I (*p* < 0.01), and Elusimicrobiota (*p* < 0.05) were higher in cbl yaks (Figure 7).

**Figure 8:**
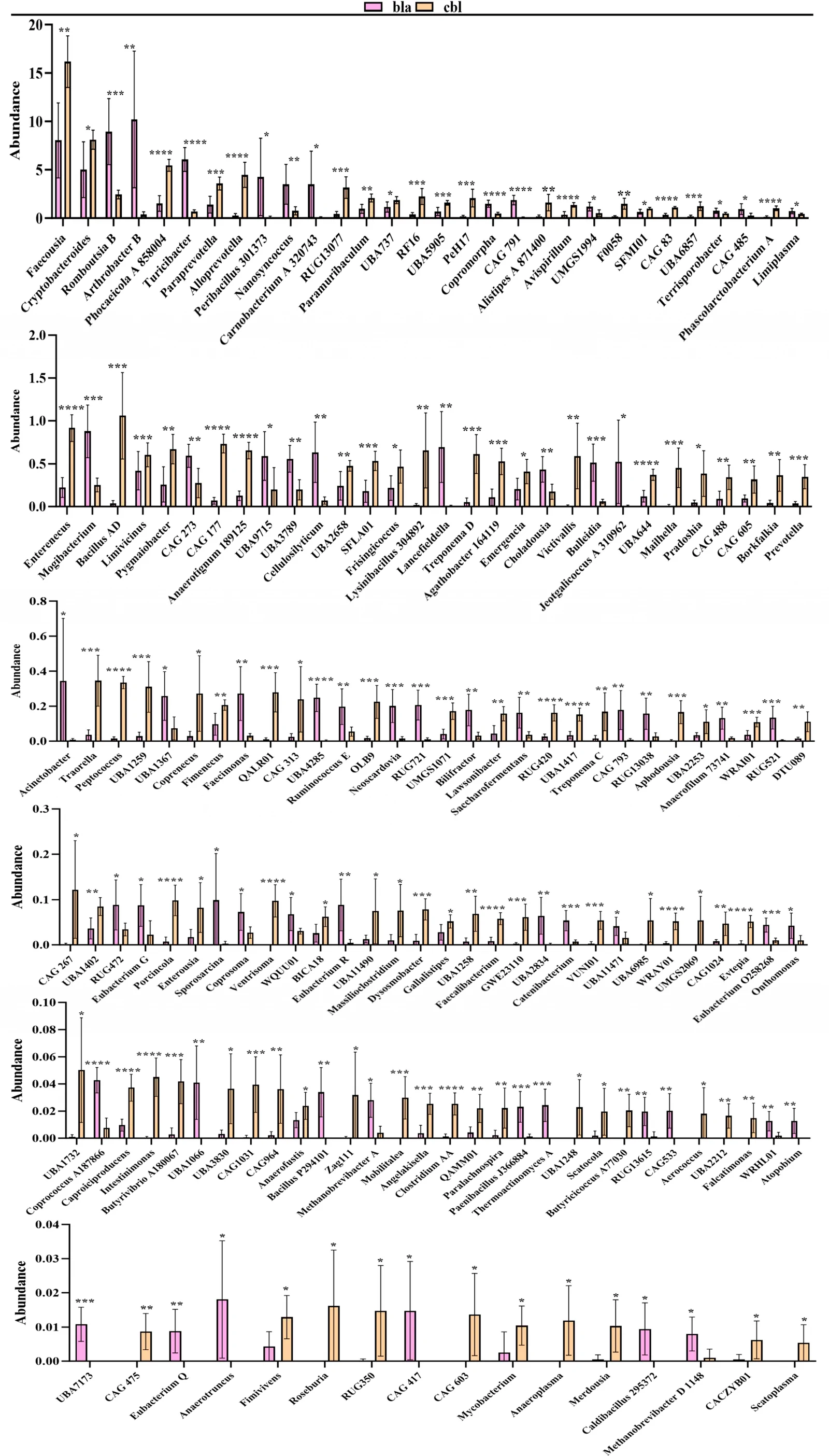
Relative abundances of bacterial genera differing between *Blastocystis*-positive and *Blastocystis*-negative diarrheic yaks. Bla denotes *Blastocystis*-positive diarrheic yaks, and cbl denotes *Blastocystis*-negative diarrheic yaks (*n* = 6 per group). Bars represent the mean ± SEM, and points represent individual animals. The *y*-axis shows relative abundance as a percentage of total bacterial sequences. Between-group differences were evaluated using an unpaired Student’s *t*-test or Mann–Whitney *U* test, as appropriate. * *p* < 0.05, ** *p* < 0.01, *** *p* < 0.001, and **** *p* < 0.0001.

At the genus level, bla yaks had higher relative abundances of *Romboutsia_B* (*p* < 0.001), *Arthrobacter_B* (*p* < 0.01), *Turicibacter* (*p* < 0.0001), *Copromorpha* (*p* < 0.0001), and CAG_791 (*p* < 0.0001). Cbl yaks had higher relative abundances of *Faecousia* (*p* < 0.01), *Cryptobacteroides* (*p* < 0.05), *Phocaeicola_A_858004* (*p* < 0.0001), *Paraprevotella* (*p* < 0.001), and *Alloprevotella* (*p* < 0.0001). Several additional genera, including *Victivallis* (*p* < 0.01), *Faecalibacterium* (*p* < 0.0001), *Caproiciproducens* (*p* < 0.0001), *Intestinimonas* (*p* < 0.0001), *Angelakisella* (*p* < 0.001), *Lawsonibacter* (*p* < 0.01), and *Roseburia* (*p* < 0.05), were also less abundant in the bla group (Figure 8).

### 3.4. Microbial Co-Occurrence Patterns and Predicted Functional Profiles

**Figure 9:**
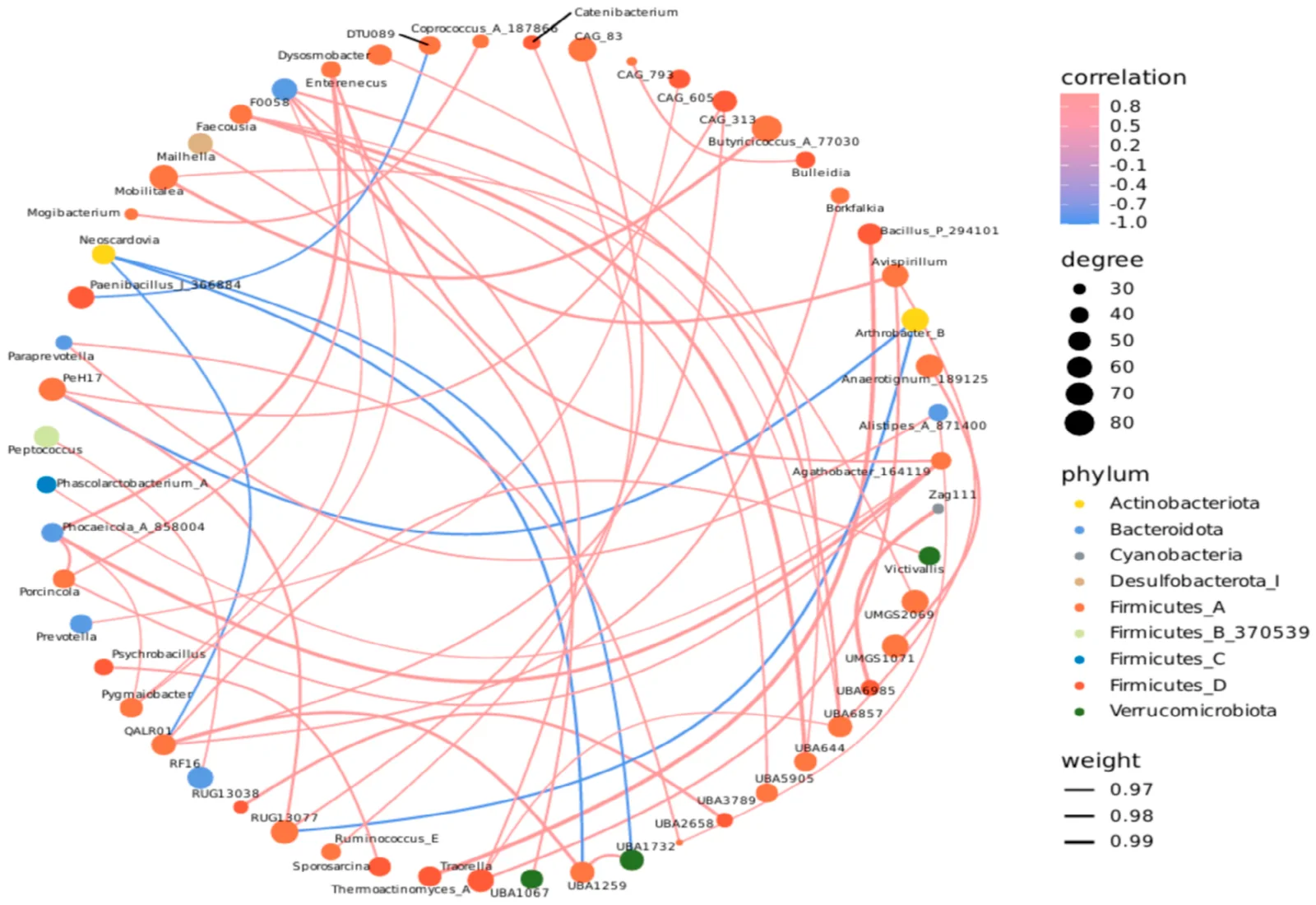
Exploratory genus-level bacterial co-occurrence network in fecal samples from diarrheic yaks. Each node represents a bacterial genus, node color indicates phylumlevel classification, and node size is proportional to degree, defined as the number of connections. Red edges indicate positive correlations, blue edges indicate negative correlations, and edge thickness represents correlation magnitude. Network edges represent statistical associations and should not be interpreted as direct ecological interactions.

Exploratory co-occurrence analysis identified several positive and negative correlations among bacterial genera. *Enterenecus* was positively correlated with *Phocaeicola_A_858004*, *Porcincola*, *Agathobacter_164119*, and *Pygmaiobacter*. *Faecousia* was positively correlated with UBA644, UBA5905, and UBA6857, whereas *Neoscardovia* was negatively correlated with QALR01, UBA1732, and UBA1259 (Figure 9). These correlations represent statistical co-occurrence patterns and do not demonstrate direct ecological interactions.

**Figure 10:**
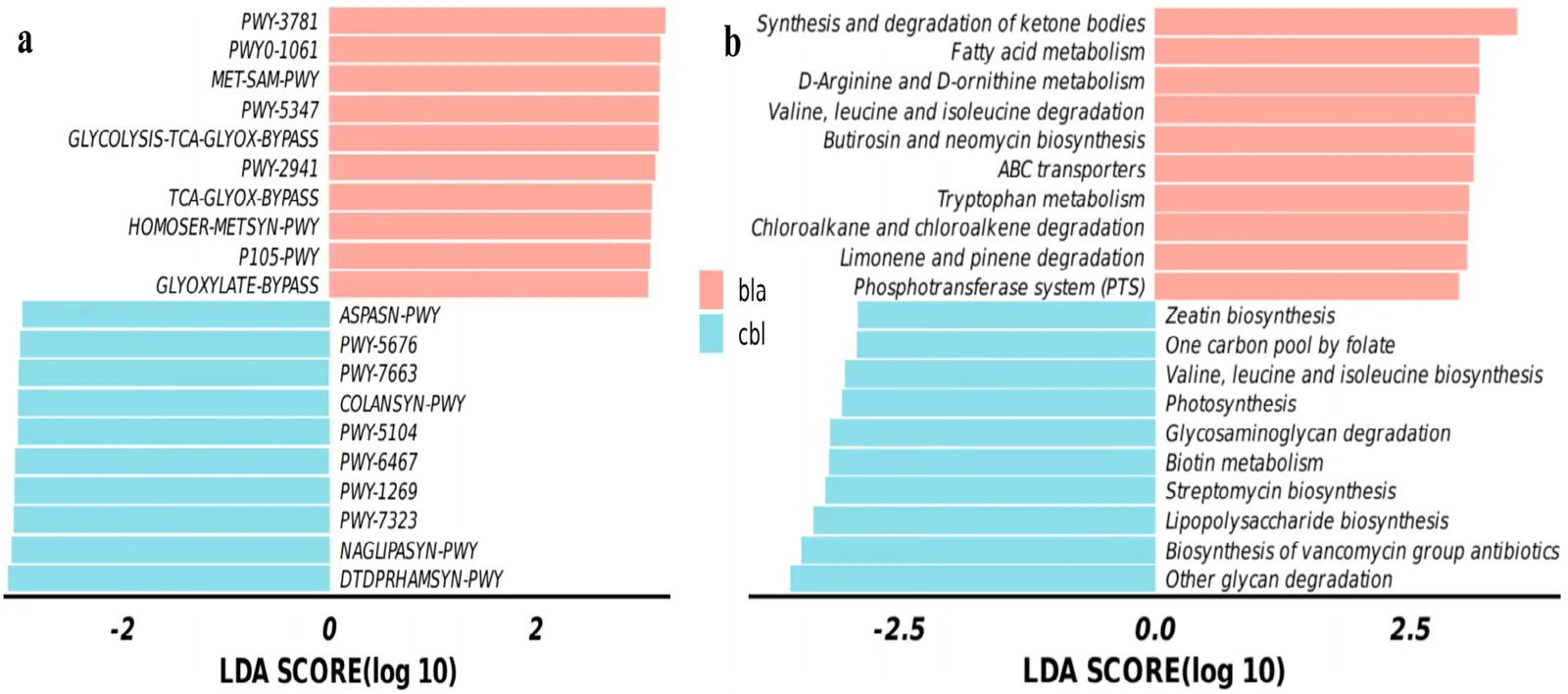
PICRUSt2-predicted microbial functional profiles in *Blastocystis*-positive and *Blastocystis*-negative diarrheic yaks. Bla denotes *Blastocystis*-positive diarrheic yaks, and cbl denotes *Blastocystis*-negative diarrheic yaks (*n* = 6 per group). Differentially represented predicted pathways are shown for (**a**) MetaCyc and (**b**) KEGG annotations. Bar color indicates the group with greater predicted pathway representation, and bar length indicates the linear discriminant analysis effect size. These pathways were inferred from 16S rRNA gene profiles and should not be interpreted as direct measurements of gene expression, enzyme activity, metabolic flux, or SCFA production.

PICRUSt2 analysis indicated differences in predicted functional potential between the groups. Predicted MetaCyc pathways enriched in the bla group included pathways related to glycolysis, the tricarboxylic acid cycle and glyoxylate bypass, methionine and homoserine metabolism, and related energy-processing functions. These included GLYCOLYSIS-TCA-GLYOX-BYPASS, TCA-GLYOX-BYPASS, GLYOXYLATE-BYPASS, MET-SAM-PWY, and HOMOSER-METSYN-PWY. Predicted pathways enriched in the cbl group included ASPASN-PWY and pathways related to cell-envelope and glycan biosynthesis, including COLANSYN-PWY and NAGLIPASYN-PWY (Figure 10a).

KEGG-based predictions indicated higher representation in bla yaks of pathways associated with ketone-body synthesis and degradation, fatty acid metabolism, branched-chain amino acid degradation, ABC transporters, the phosphotransferase system, and tryptophan metabolism. In cbl yaks, predicted pathways related to branched-chain amino acid biosynthesis, the one-carbon pool by folate, glycosaminoglycan degradation, biotin metabolism, and lipopolysaccharide biosynthesis were more highly represented (Figure 10b).

## 4. Discussion

This exploratory cross-sectional study found that *Blastocystis*-positive diarrheic yaks had lower fecal concentrations of six SCFAs, lower bacterial richness and diversity, different bacterial community composition, and differences in several taxa and predicted microbial functions compared with *Blastocystis*-negative diarrheic yaks. Because both groups consisted of diarrheic animals, these findings represent associations with *Blastocystis* status within a diarrheic population and do not demonstrate that *Blastocystis* caused the observed microbial or metabolic differences.

ACE, Chao1, Faith’s phylogenetic diversity, and observed-species values were approximately 29–30% lower in the bla group, while Shannon diversity was approximately 18% lower. The cbl group also contained more group-specific ASVs. Comparable Good’s coverage and the rarefaction patterns supported adequate characterization of the observed bacterial communities. PCA, PCoA, NMDS, UPGMA, and ANOSIM further indicated differences in community composition between the groups. However, the relationship between *Blastocystis* and bacterial diversity differs among hosts and subtypes. Higher diversity has been reported in some asymptomatic human carriers [12,13], whereas ST7-positive diarrheic patients showed lower diversity and altered bacterial profiles [14]. Subtype-dependent associations have also been reported in forest musk deer [15]. Experimental ST1 colonization in mice was associated with changes in microbial composition and immune responses [16], whereas chronic ST4 colonization in rats was accompanied by altered microbiota and lower fecal SCFAs [17]. Helminth-associated microbiota changes may involve different type-2 and regulatory immune pathways [26]. These findings suggest that associations between *Blastocystis* and the microbiota may vary according to subtype, host species, clinical condition, diet, background microbiota, and environment. The reduced alpha diversity observed in *Blastocystis*-positive diarrheic yaks in the present study may reflect the specific host and environmental context. Unlike several human cohort studies involving asymptomatic carriers, the current study included only diarrheic animals, in which the intestinal ecosystem was likely already disturbed. Therefore, Blastocystis colonization may interact with pre-existing intestinal instability and contribute to further reductions in microbial community complexity. In addition, plateau yaks experience unique environmental pressures, including chronic hypoxia, cold exposure, seasonal nutritional fluctuations, and reliance on high-fiber forage, which may influence microbial adaptation and intestinal resilience. Although the present study cannot directly demonstrate an interaction between hypoxia and *Blastocystis* colonization, these high-altitude characteristics may represent important factors affecting host–microbiota–protist relationships.

Plateau conditions may also influence the observed relationships. Yak gastrointestinal microbiota varies with geographical location, age, sex, feeding strategy, and gastrointestinal region [27], while diarrhea itself can alter bacterial and fungal communities [2]. Microbial fermentation contributes substantially to the utilization of fibrous forage and the adaptation of yaks to high-altitude conditions [28]. These characteristics provide a basis for investigating whether the plateau environment modifies protist–microbiota relationships. However, altitude gradients, dietary intake, oxygen-related responses, and intestinal immune indicators were not measured; therefore, an interaction between hypoxia and *Blastocystis* cannot be concluded from the present data.

Acetic, propionic, butyric, valeric, caproic, and isobutyric acids were lower in bla yaks, whereas isovaleric acid did not differ significantly. Fecal SCFA concentrations reflect the combined effects of microbial formation, intestinal absorption, host utilization, transit time, fecal water content, and substrate availability and are not direct measurements of microbial production. Nevertheless, the concurrent reduction in several SCFAs was consistent with differences in fermentation-related microbial metabolism. This pattern was directionally consistent with the lower relative abundance of Bacteroidota and of genera previously associated with carbohydrate fermentation or SCFA formation, including *Alloprevotella*, *Faecalibacterium*, *Caproiciproducens*, *Intestinimonas*, *Angelakisella*, and *Roseburia*. Such functions can vary among species and strains and cannot be confirmed from V3–V4 amplicon data alone. SCFAs participate in epithelial and immune regulation [4], and GPR43-dependent signaling can influence antimicrobial peptide expression through mTOR and STAT3 [29]. Lower fecal SCFA concentrations may therefore be relevant to intestinal homeostasis, but permeability, tight-junction proteins, GPR43 expression, cytokines, and histopathology were not evaluated. SCFA depletion may represent a potential link connecting microbial dysbiosis with impaired intestinal function. Butyrate is an important energy source for intestinal epithelial cells and contributes to epithelial barrier maintenance, whereas acetate and propionate can regulate host metabolism and immune responses through SCFA receptors, including GPR43 (FFAR2) [30,31]. Reduced SCFA availability may therefore influence epithelial homeostasis and inflammatory balance. In the context of plateau yaks, environmental stressors associated with high altitude may further challenge intestinal adaptation, potentially increasing the vulnerability of animals with disturbed microbial fermentation. Thus, we propose a hypothetical pathway in which *Blastocystis* positivity is associated with altered bacterial communities, reduced SCFA-producing capacity, decreased SCFA availability, and impaired intestinal homeostasis. However, direct evidence for this mechanism requires future studies measuring SCFA-related signaling pathways, epithelial barrier markers, and immune responses.

Bacteroidota decreased from 35.19% in cbl yaks to 12.53% in bla yaks, whereas Firmicutes_D increased from 5.23% to 23.36%. Verrucomicrobiota, Spirochaetota, Firmicutes_C, Firmicutes_B_370539, and Desulfobacterota_I were also less abundant in bla yaks. The yak rumen microbiome contains extensive carbohydrate-active enzyme capacity that contributes to fiber degradation [32]. The observed fecal taxonomic differences may therefore be relevant to fiber-associated microbial metabolism, although their consequences for forage digestibility, energy supply, growth, or productivity were not measured. Furthermore, fecal bacterial communities do not directly represent ruminal fermentation. The higher Firmicutes-to-Bacteroidota ratio in bla yaks should not be considered a universal marker of dysbiosis because this ratio varies with host species, diet, gastrointestinal location, sample processing, sequencing method, and disease context [33].

LEfSe and exploratory comparisons showed greater relative representation of Firmicutes_D, Actinobacteriota, Bacilli, *Arthrobacter_B*, *Romboutsia_B*, *Turicibacter*, and *Copromorpha* in Bla yaks, whereas Bacteroidota, Bacteroidia, *Faecousia*, *Cryptobacteroides*, *Phocaeicola_A_858004*, *Paraprevotella*, and *Alloprevotella* were more represented in cbl yaks. These taxa should be described as group-associated microbial features rather than diagnostic biomarkers because diagnostic performance and external reproducibility were not assessed. Similarly, the greater relative abundance of *Acinetobacter* or other genera previously associated with disease does not demonstrate pathogenic activity, because biological properties vary among species and strains. The findings must also be interpreted as compositional relative-abundance data; an apparent increase in one taxon can result from decreases in other taxa without an increase in absolute bacterial abundance [34]. Because numerous taxa were compared, findings not retained after false-discovery-rate correction should be regarded as exploratory [25].

PICRUSt2 predicted differences in pathways related to carbohydrate metabolism, glycolysis, the tricarboxylic acid and glyoxylate cycles, fatty acid metabolism, amino acid metabolism, ABC transporters, the phosphotransferase system, one-carbon metabolism, biotin metabolism, and glycan degradation. The presence of lower measured SCFAs together with greater predicted representation of some energy-metabolism pathways in bla yaks may reflect differences between predicted genomic potential and actual metabolic activity. PICRUSt2 infers functional potential from 16S rRNA profiles and reference genomes and does not directly measure genes, transcription, enzymes, pathway flux, or metabolite formation [22]. These predictions therefore require validation using shotgun metagenomics, metatranscriptomics, or metabolomics. The microbial network similarly showed exploratory co-occurrence patterns involving *Enterenecus*, *Faecousia*, and *Neoscardovia*. These correlations may reflect shared environmental responses, indirect relationships, or compositional effects and should not be interpreted as direct microbial interactions.

Previous yak studies reported ST10, ST12, ST14, ST21, ST24, ST25, ST26, ST42, and ST44, with ST10 frequently detected in ruminants [8,10]. In the present study, the sequences were confirmed as *Blastocystis* spp., but definitive subtype assignment was unavailable. Consequently, the microbiota and SCFA findings cannot be attributed to a specific subtype or used to infer zoonotic transmission. A study from Lhasa detected *Blastocystis* together with *Cryptosporidium*, *Giardia intestinalis*, and *Enterocytozoon bieneusi* in yak populations [11], indicating that exposure to multiple enteric organisms is possible. Altered bacterial communities and lower SCFA concentrations have also been reported in *Cryptosporidium*-positive plateau yaks and Tibetan pigs [35,36], but these similarities do not establish a common mechanism. Other pathogens were not systematically excluded in the present study and may have influenced the results. Evidence of zoonotic transmission would require subtype-resolved comparison of isolates collected concurrently from yaks, humans, other livestock, water, soil, and shared environments.

From a veterinary perspective, *Blastocystis* testing may form part of broader investigations of diarrhea in yaks, but PCR positivity alone does not identify the cause of diarrhea or provide a basis for specific treatment. Clinical assessment should also consider diet, hydration, medication history, diarrhea severity, and other protozoal, bacterial, viral, and helminth infections. General biosecurity measures, including appropriate fecal management and protection of feed and water from fecal contamination, may reduce opportunities for fecal–oral exposure. Dietary or microbiota-directed strategies may warrant investigation because herbal formulations have been associated with changes in yak microbiota, antioxidant indicators, and inflammatory mediators [5,37]. However, probiotics, prebiotics, SCFA supplementation, and herbal formulations have not been validated for *Blastocystis*-associated disease in yaks and should not be presented as established interventions.

Several limitations should be considered when interpreting these findings. The study was cross-sectional, included only six animals per group, and was conducted in free-ranging yaks from a single farm within one geographical region. Sampling from a single farm reduced between-farm environmental heterogeneity but limits the generalizability of the findings to yaks from other herds, geographical areas, and management systems. Although 11 of the 30 diarrheic fecal samples screened positive for *Blastocystis* spp., only six yielded successful sequence confirmation and were included in the downstream comparative analyses; therefore, selection related to successful sequencing cannot be excluded. All included animals were diarrheic, and no non-diarrheic healthy comparison group was available. Complete animal-level information on age, sex, body condition, detailed dietary intake, vaccination history, recent antimicrobial or antiparasitic treatment, and diarrhea severity was unavailable, preventing individual matching or statistical adjustment for these variables. Although both groups originated from the same farm and shared the same general free-ranging management system, residual host, dietary, clinical, and management confounding cannot be excluded. Systematic screening for alternative bacterial, viral, protozoal, and helminth causes of diarrhea was not performed; consequently, concurrent enteric infections may have influenced the bacterial-community and SCFA profiles.

The final sample size was small, and no a priori power calculation was performed, reducing statistical power and increasing sensitivity to individual variation. The microbiome analyses were based predominantly on relative-abundance data, and apparent changes in individual taxa may partly reflect the compositional nature of sequencing data rather than changes in absolute bacterial abundance. The correlation-based microbial network should similarly be regarded as exploratory because stable network inference is difficult with a limited number of samples. PICRUSt2 results represent computational predictions of microbial functional potential rather than direct measurements of genes, gene expression, enzymes, metabolic flux, or metabolite production. Fecal SCFA concentrations reflect the combined effects of microbial production, intestinal absorption, host utilization, transit time, fecal water content, and substrate availability and therefore do not directly quantify intestinal SCFA production. In addition, absolute bacterial loads, fungi, viruses, host immune responses, epithelial integrity, digestibility, and production traits were not measured. Finally, definitive *Blastocystis* subtype assignment was unavailable; therefore, subtype-specific pathogenic effects or zoonotic transmission cannot be inferred from the present study. Larger longitudinal and multi-farm studies incorporating clinically healthy and diarrheic comparison groups, detailed animal and dietary metadata, comprehensive enteric-pathogen screening, subtype-resolved *Blastocystis* analysis, compositional microbiome methods, and direct host and microbial functional measurements are required to validate these associations.

## 5. Conclusions

This exploratory study showed that *Blastocystis*-positive diarrheic yaks had lower fecal concentrations of several SCFAs, reduced bacterial diversity, and distinct fecal bacterial-community profiles compared with *Blastocystis*-negative diarrheic yaks. These findings indicate an association between *Blastocystis* positivity, altered bacterial communities, and reduced fecal SCFA concentrations. Because the study was cross-sectional, included only six animals per group, and involved exclusively diarrheic yaks, causal relationships cannot be established. Larger controlled studies incorporating healthy comparison groups, comprehensive co-infection screening, *Blastocystis* subtype identification, and host-health measurements are required to determine the biological and veterinary significance of these associations.

## Figures and Tables

**Table 1:** Sequencing information of *Blastocystis* spp.-positive and *Blastocystis* spp.-negative yak fecal samples.

Samples	Input	Filtered	Denoised	Merged	Non-Chimeric	Non-Singleton
bla-1	255,925	243,682	240,551	225,370	219,082	219,019
bla-2	249,898	237,824	234,227	216,906	211,437	211,365
bla-3	228,898	218,153	214,345	192,581	185,426	185,308
bla-4	176,917	168,618	165,559	148,936	144,125	144,065
bla-5	203,878	194,351	191,567	180,375	171,956	171,898
bla-6	188,208	179,554	176,754	164,007	156,916	156,834
cbl-1	197,284	187,962	184,784	166,016	160,289	160,229
cbl-2	204,935	194,928	191,118	170,216	165,042	164,954
cbl-3	201,073	191,648	188,259	170,663	165,544	165,464
cbl-4	228,645	217,790	213,748	190,614	183,081	183,024
cbl-5	334,895	319,023	312,290	266,959	254,345	254,134
cbl-6	280,472	267,416	262,860	232,787	219,867	219,752

**Table 2:** Alpha diversity indices of bacterial communities in *Blastocystis* spp.-positive and *Blastocystis* spp.-negative diarrheic yaks.

Sample	Chao1	ACE	Faith’s pd	Good’s Coverage	ASVs	Pielou’s e	Shannon	Simpson
bla-1	2270.94	2263.60	59.66	0.999	2239	0.766	8.53	0.986
bla-2	2417.81	2416.82	76.93	0.999	2403	0.805	9.04	0.991
bla-3	2370.5	2371.28	78.10	0.999	2351	0.803	9.002	0.990
bla-4	2279.87	2281.81	60.26	0.999	2277	0.840	9.37	0.992
bla-5	1423.97	1426.45	48.62	0.999	1416	0.638	6.68	0.959
bla-6	1722.39	1724.16	56.81	0.999	1718	0.677	7.27	0.967
cbl-1	2701.83	2703.10	91.57	0.999	2694	0.893	10.18	0.998
cbl-2	2948.39	2951.06	96.53	0.999	2942	0.892	10.27	0.997
cbl-3	2686.01	2686.35	87.25	0.999	2676	0.877	9.98	0.997
cbl-4	2845.88	2848.45	96.49	0.999	2835	0.888	10.18	0.997
cbl-5	3942.68	3929.32	90.41	0.999	3889	0.884	10.54	0.998
cbl-6	2673.50	2669.24	72.28	0.999	2646	0.858	9.76	0.996

## Data Availability

The raw 16S rRNA gene sequencing data are available in the NCBI Sequence Read Archive under BioProject accession PRJNA1414128. The *Blastocystis* SSU rRNA gene sequences are available in GenBank under accession numbers PX888047–PX888052.

## References

[1] Wang X., Pei J., Xiong L., Bao P., Chu M., Ma X., La Y., Liang C., Yan P., Guo X. (2024). Genetic diversity, phylogeography, and maternal origin of yak (*Bos grunniens*). BMC Genom..

[2] Wang D., Zeng J., Ma H., Rasheed M., Su Z., Mohamed R.A.E.H., Ibrahim E.H. (2025). Microbiome analysis shows the adverse impacts of diarrhea on the intestinal homeostasis in yaks. Pak. Vet. J..

[3] Khan M.T., Zohair M., Khan A., Kashif A., Mumtaz S., Muskan F. (2025). From gut to brain: The roles of intestinal microbiota, immune system, and hormones in intestinal physiology and the gut–brain axis. Mol. Cell. Endocrinol..

[4] Zhang D., Jian Y.P., Zhang Y.N., Li Y., Gu L.T., Sun H.H., Liu M.D., Zhou H.L., Wang Y.S., Xu Z.X. (2023). Short-chain fatty acids in diseases. Cell Commun. Signal..

[5] Zhu Y., Lu S., Cidan Y., Ali M., Zhang X., Pubu P., Kiani F.A., Saleem M.U., Basang W., Li K. (2025). Protective effects of traditional Chinese herbal medicine formulas via influencing antioxidative capacity, inflammatory mediators, and gut microbiota in weaned yaks. Pak. Vet. J..

[6] Aykur M., Malatyalı E., Demirel F., Cömert-Koçak B., Gentekaki E., Tsaousis A.D., Dogruman-Al F. (2024). *Blastocystis*: A mysterious member of the gut microbiome. Microorganisms.

[7] Rehena J., Bin Harun A., Karim M.R. (2025). Epidemiology of *Blastocystis* in farm animals: A review. Vet. Parasitol..

[8] Ren M., Song J.K., Yang F., Zou M., Wang P.X., Wang D., Zhang H.J., Zhao G.H., Lin Q. (2019). First genotyping of *Blastocystis* in yaks from Qinghai Province, northwestern China. Parasites Vectors.

[9] Chen X. (2022). Molecular epidemiological investigation of *Cryptosporidium* sp., *Giardia duodenalis*, *Enterocytozoon bieneusi*, and *Blastocystis* sp. infection in free-ranged yaks and Tibetan pigs on the plateau. Pak. Vet. J..

[10] Zhao H., Ma W., Zhang B., He Y., Zhang Z., Zhao A., Yu F., Qi M. (2025). Genetic diversity and host specificity of *Blastocystis* in yaks in Xinjiang, northwest China. Acta Trop..

[11] Ji Y., Ali M., Xu C., Wang J., Kulyar F., Nawaz S., Mehmood K., Liu M., Li K. (2025). Epidemiological exploration of *Cryptosporidium* spp., *Giardia intestinalis*, *Enterocytozoon bieneusi*, and *Blastocystis* spp. in yaks: Investigating ecological and zoonotic dynamics in Lhasa, Xizang. Vet. Sci..

[12] Stensvold C.R., Sørland B.A., Berg R.P.K.D., Andersen L.O., van der Giezen M., Bowtell J.L., El-Badry A.A., Belkessa S., Kurt Ö., Nielsen H.V. (2022). Stool microbiota diversity analysis of *Blastocystis*-positive and *Blastocystis*-negative individuals. Microorganisms.

[13] Antonetti L., Berrilli F., Di Cristanziano V., Farowski F., Daeumer M., Eberhardt K.A., Santoro M., Federici M., D’aLfonso R. (2024). Investigation of gut microbiota composition in humans carrying *Blastocystis* subtypes 1 and 2 and *Entamoeba hartmanni*. Gut Pathog..

[14] Deng L., Lee J.W.J., Tan K.S.W. (2022). Infection with pathogenic *Blastocystis* ST7 is associated with decreased bacterial diversity and altered gut microbiome profiles in diarrheal patients. Parasites Vectors.

[15] Deng L., Chen S., Meng W., Zhou Z., Liu H., Zhong Z., Fu H., Shen L., Cao S., Tan K.S.W. (2022). Changes in gut microbiota composition associated with the presence of enteric protist *Blastocystis* in captive forest musk deer (*Moschus Berezovskii*). Microbiol. Spectr..

[16] Deng L., Wojciech L., Png C.W., Kioh Y.Q.D., Ng G.C., Chan E.C.Y., Zhang Y., Gascoigne N.R.J., Tan K.S.W. (2023). Colonization with ubiquitous protist *Blastocystis* ST1 ameliorates DSS-induced colitis and promotes beneficial microbiota and immune outcomes. npj Biofilms Microbiomes.

[17] Defaye M., Nourrisson C., Baudu E., Lashermes A., Meynier M., Meleine M., Wawrzyniak I., Bonnin V., Barbier J., Chassaing B. (2020). Fecal dysbiosis associated with colonic hypersensitivity and behavioral alterations in chronically *Blastocystis*-infected rats. Sci. Rep..

[18] Bolyen E., Rideout J.R., Dillon M.R., Bokulich N.A., Abnet C.C., Al-Ghalith G.A., Alexander H., Alm E.J., Arumugam M., Asnicar F. (2019). Reproducible, interactive, scalable and extensible microbiome data science using QIIME 2. Nat. Biotechnol..

[19] Callahan B.J., Mcmurdie P.J., Rosen M.J., Han A.W., Johnson A.J.A., Holmes S.P. (2016). DADA2: High-resolution sample inference from Illumina amplicon data. Nat. Methods.

[20] Katoh K., Standley D.M. (2013). MAFFT multiple sequence alignment software version 7: Improvements in performance and usability. Mol. Biol. Evol..

[21] Segata N., Izard J., Waldron L., Gevers D., Miropolsky L., Garrett W.S., Huttenhower C. (2011). Metagenomic biomarker discovery and explanation. Genome Biol..

[22] Douglas G.M., Maffei V.J., Zaneveld J.R., Yurgel S.N., Brown J.R., Taylor C.M., Huttenhower C., Langille M.G.I. (2020). PICRUSt2 for prediction of metagenome functions. Nat. Biotechnol..

[23] Zhu J.H., Mao Q., Wang S.Y., Liu H., Zhou S.S., Zhang W., Kong M., Zhu H., Li S.L. (2022). Optimization and validation of direct gas chromatography–mass spectrometry method for simultaneous quantification of ten short-chain fatty acids in rat feces. J. Chromatogr. A.

[24] Kim K.S., Lee Y., Chae W., Cho J.Y. (2022). An improved method to quantify short-chain fatty acids in biological samples using gas chromatography–mass spectrometry. Metabolites.

[25] Benjamini Y., Hochberg Y. (1995). Controlling the false discovery rate: A practical and powerful approach to multiple testing. J. R. Stat. Soc. Ser. B.

[26] Myhill L.J., Stolzenbach S., Mejer H., Krych L., Jakobsen S.R., Kot W., Skovgaard K., Canibe N., Nejsum P., Nielsen D.S. (2022). Parasite–probiotic interactions in the gut: *Bacillus* sp. and *Enterococcus faecium* regulate type-2 inflammatory responses and modify the gut microbiota of pigs during helminth infection. Front. Immunol..

[27] Su Y., Su J., Li F., Tian X., Liu Z., Ding G., Bai J., Li Z., Ma Z., Peppelenbosch M.P. (2022). Yak gut microbiota: A systematic review and meta-analysis. Front. Vet. Sci..

[28] Huang C., Zhang M., Zheng Q., Yu Q., Yang G., Ren W., Ma X., La Y., Bao P., Chu M. (2026). The high-altitude adaptation characteristics of microbiota–host cross-talk in yak gastrointestinal track. Adv. Sci..

[29] Zhao Y., Chen F., Wu W., Sun M., Bilotta A.J., Yao S., Xiao Y., Huang X., Eaves-Pyles T.D., Golovko G. (2018). GPR43 mediates microbiota metabolite SCFA regulation of antimicrobial peptide expression in intestinal epithelial cells via activation of mTOR and STAT3. Mucosal Immunol..

[30] Donohoe D.R., Garge N., Zhang X., Sun W., O’Connell T.M., Bunger M.K., Bultman S.J. (2011). The microbiome and butyrate regulate energy metabolism and autophagy in the mammalian colon. Cell Metab..

[31] Kim C.H., Park J., Kim M. (2014). Gut microbiota-derived short-chain fatty acids, T cells, and inflammation. Immune Netw..

[32] Zhao C., Wang L., Ke S., Chen X., Kenéz Á., Xu W., Wang D., Zhang F., Li Y., Cui Z. (2022). Yak rumen microbiome elevates fiber degradation ability and alters rumen fermentation pattern to increase feed efficiency. Anim. Nutr..

[33] Magne F., Gotteland M., Gauthier L., Zazueta A., Pesoa S., Navarrete P., Balamurugan R. (2020). The Firmicutes/Bacteroidetes ratio: A relevant marker of gut dysbiosis in obese patients?. Nutrients.

[34] Gloor G.B., Macklaim J.M., Pawlowsky-Glahn V., Egozcue J.J. (2017). Microbiome datasets are compositional: And this is not optional. Front. Microbiol..

[35] Dong H., Chen X., Zhao X., Zhao C., Mehmood K., Kulyar M.F.E.A., Bhutta Z.A., Zeng J., Nawaz S., Wu Q. (2023). Intestine microbiota and SCFAs response in naturally *Cryptosporidium*-infected plateau yaks. Front. Cell. Infect. Microbiol..

[36] Chen X., Zhao X., Zhao C., Ashfaq H., Kulyar M.F.-E., Bhutta Z.A., Ali M.M., Mansoor M.K., Li K. (2023). *Cryptosporidium* infection induced the dropping of SCFAs and dysbiosis in the intestinal microbiome of Tibetan pigs. Microb. Pathog..

[37] Cidan Y., Cisang Z., Tian J., Zhang X., Safdar M., Pubu P., Zhu Y., Basang W. (2025). Tibetan herbal formulations enhanced the antioxidant capacity of weaned female yaks by modulating the gut microbiota. Pak. Vet. J..

